# Mining, compressing and classifying with extensible motifs

**DOI:** 10.1186/1748-7188-1-4

**Published:** 2006-03-23

**Authors:** Alberto Apostolico, Matteo Comin, Laxmi Parida

**Affiliations:** 1 Dipartimento di Ingegneria dell'lnformazione, Università di Padova, Padova, Italy; 2 College of Computing, Georgia Institute of Technology, 801 Atlantic Drive, Atlanta, GA 30332, USA; 3 IBM T. J. Watson Research Center, Yorktown Heights, NY 10598, USA

## Abstract

**Background:**

Motif patterns of maximal saturation emerged originally in contexts of pattern discovery in biomolecular sequences and have recently proven a valuable notion also in the design of data compression schemes. Informally, a motif is a string of intermittently solid and wild characters that recurs more or less frequently in an input sequence or family of sequences. Motif discovery techniques and tools tend to be computationally imposing, however, special classes of "rigid" motifs have been identified of which the discovery is affordable in low polynomial time.

**Results:**

In the present work, "extensible" motifs are considered such that each sequence of gaps comes endowed with some elasticity, whereby the same pattern may be stretched to fit segments of the source that match all the solid characters but are otherwise of different lengths. A few applications of this notion are then described. In applications of data compression by textual substitution, extensible motifs are seen to bring savings on the size of the codebook, and hence to improve compression. In germane contexts, in which compressibility is used in its dual role as a basis for structural inference and classification, extensible motifs are seen to support unsupervised classification and phylogeny reconstruction.

**Conclusion:**

Off-line compression based on extensible motifs can be used advantageously to compress and classify biological sequences.

## Background

Let *s *be a sequence of sets of characters from an alphabet Σ ⋃ {·}, where '.' ∉ Σ denotes a don't care (*dot*, for short) and the rest are *solid *characters, we use *σ *to denote a singleton character. For characters *e*_1 _and *e*_2_, we write *e*_1 _∘ *e*_2 _if and only if *e*_1 _is a dot or *e*_1 _= *e*_2_. Allowing for spacers in a string is what makes it extensible. Such spacers are indicated by annotating the dot characters. Specifically, an annotated "." character is written as .^*α *^where *α *is a set of positive integers {*α*_1_, *α*_2_, ..., *α*_*k*_} or an interval *α *= [*α*_*l*_, *α*_*u*_], representing all integers between *α*_*l *_and *α*_*u *_including *α*_*l *_and *α*_*u*_. Whenever defined, *d *will denote the maximum number of consecutive dots allowed in a string. In such cases, for clarity of notation, we use the *extensible wild card *denoted by the dash symbol "-" instead of the annotated dot character, .^[1,*d*] ^in the string. Note that '-' ∉ Σ. Thus a string of the form *a*.^[1,*d*]^*b *will be simply written as *a*-*b*. A motif *m *is *extensible *if it contains at least one annotated dot, otherwise *m *is *rigid*. Given an extensible string *m*, a rigid string *m' *is a *realization *of *m *if each annotated dot .^*α *^is replaced by *l *∈ *α *dots. The collection of all such rigid realizations of *m *is denoted by *R*(*m*). A rigid string *m *occurs at position *l *on *s *if *m*[*j*] ∘ *s*[*l *+ *j *- 1] holds for 1 ≤ *j *≤ |*m*|. An extensible string *m *occurs at position *l *in *s *if there exists a realization *m' *of *m *that occurs at *l*. Note than an extensible string *m *could possibly occur more than once at a location on a sequence *s*. Throughout in the discussion we are interested mostly in the (unique) first left-most occurrence at each location.

For a sequence *s *and positive integer *k*, *k *≤ |*s*|, a string (extensible or rigid) *m *is a *motif *of *s *with |*m*| > 1 and location list _*m *_= (*l*_1_, *l*_2_, ..., *l*_*p*_), if both *m*[1] and *m*[|*m*|] are solid and _*m*_, |_*m*_| ≥ *k*, is the list of all and only the occurrences of *m *in *s*. Given a motif *m *let *m*[*j*_1_], *m*[*j*_2_], ... *m*[*j*_*l*_] be the *l *solid elements in the motif *m*. Then the sub-motifs of *m *are given as follows: for every *j*_*i*_, *j*_*t*_, the sub-motif *m*[*j*_*i *_... *j*_*t*_] is obtained by dropping all the elements before (to the left of) *j*_*i *_and all elements after (to the right of) *j*_*t *_in *m*. We also say that *m *is a *condensation *for any of its sub-motifs. We are interested in motifs for which any condensation would disrupt the list of occurrences. Formally, let *m*_1_, *m*_2_, ..., *m*_*j *_be the motifs in a string *s*. A motif *m*_*i *_is *maximal in length *if there exists no *m*_*l*_, *l *≠ *i *with  and *m*_*i *_is a sub-motif of *m*_*l*_. A motif *m*_*i *_is *maximal in composition *if no dot character of *m*_*i *_can be replaced by a solid character that appears in all the locations in _*m*_. A motif *m*_*i *_is *maximal in extension *if no annotated dot character of *m*_*i *_can be replaced by a fixed length substring (without annotated dot characters) that appears in all the locations in _*m*_. A maximal motif is maximal in composition, in extension and in length. For an exhaustive description of these properties we refer the reader to [1].

## Results and discussion

Several measures of distance have been proposed and used to classify documents of diverse nature and to infer relationships among them. In practice, each measure translates in a computational task which might be more or less of a burden. In domains such as genome analysis and natural language processing, the increasing availability of longer and longer sequences and more and more massive data sets is playing havoc with similarity measures based on edit computations and the likes [2]. As an alternative, succinct scores related to compressibility -interpreted as a measure of structural complexity or information contents- have been deployed, of which the lineage may be traced back to Kolmogorov's complexity. The *Kolmogorov complexity *of a string *x*, denoted *K*(*x*), is the length of the shortest program that would cause a standard universal computer to output *x*. Along the same lines, the *conditional Kolmogorov complexity K*(*x*|*y*) for strings *x *and *y *is defined as the length of the shortest program that, given *y *as input, will output *x *as the result. Intuitively, the conditional complexity expresses the information difference between the strings *x *and *y*. We refer the reader to, e.g., [3] for a detailed treatment of the theory. Whereas the original Kolmogorov complexity is hardly computable, important emulators have been developed since [4], which conjugate compressibility and ease of computation. Following in these steps, we now test the discriminating power of the data compression method that is based on our Off-line steepest descent paradigm with extensible motifs.

In this paper, we present lossy off-line data compression techniques by textual substitution in which the patterns used in compression are chosen among the extensible motifs that are found to recur in the textstring with a minimum pre-specified frequency. Motif discovery and motif-driven parses of various kinds have been previously introduced and used in [5]. Whereas the motifs considered in those studies are "rigid", here we assume that each sequence of gaps present in a motif comes endowed with some individually prescribed degree of elasticity, whereby a same pattern may be stretched to fit segments of the source that match all the solid characters but are otherwise of different lengths. This is expected to save on the size of the codebook, and hence to improve compression.

The figure of compression achieved by our algorithm shows good sensitivity in telling apart veritable families of proteins from spurious blends. This sets forth an approach to classification that does away with alignment. The data used for the test consists of protein sequences, which are known to be hardly compressible at all [6]. The experiment reported below uses three different families which were picked at random from the PROSITE repository: AP endonucleases (acnucl), G-protein coupled receptors (gprot) and Succinyl-CoA ligases (succ). Table 2 summarizes the results of lossy and lossless compression for various values of the parameters. The artificial groups are marked "-mix", the last column shows the lossless compression ratio of fake over faithful families, when using motifs with the same parameter values. In all cases, the artificial families show compression ratios that are poorer by 10/20%, and the superiority of the lossy variants manifests itself throughout. The experiments thus verify the discrimination potential of data compression by extensible motifs. It seems thus meaningful to build a classifier on top of this measure. Compressibility by extensible motifs may be used to set up a similarity measure on sequences to be used in the inference of phylogeny. The measure could be extended into a metric distance, along the lines of [7]. Specifically, we denote by *Off-line*(*z*) the output size obtained when compressing a string *z *using the lossless variant of our paradigm, and compute the quantity:

**Table 1:** The pseudocode of the motif extraction algorithm.

**Main**()	**Iterate**(*m*, *B*, *Result*)
{	{
*Result *← {};	G:l *m' *← *m*;
*B *← {*m*_*i*_|*m*_*i *_is a cell};	G:2 For each *b *= *Extract*(*B*) with
For each *m *= *Extract*(*B*)	G:3 ((*b *~-*compatible m'*
**Iterate**(*m*, *B*, *Result*);	OR (*m' *~-*compatible b))*
*Result *← *Result*;	G:4 If (*m' *~-*compatible b*)
}	G:5 *m*_*t *_← *m' *~ *b*;
	G:6 If *Nodelnconsistent*(*m*_*t*_) exit;
	G:7 If (|_*m'*_| = |_*b*_|) *B *← *B *- {*b*};
	G:8 If (|| ≤ *K*)
	G:9 *m' *← *m*_*t*_;
	G:10 **Iterate**(*m'*, *B*, *Result*);
	G:11 If (*b *~-*compatible m'*)
	G:12 *m*_*t *_← *b *~ *m'*;
	G:13 If *Nodelnconsistent*(*m*_*t*_) exit;
	G:14 If (|_*m'*_| = |_*b*_|) *B *← *B *- {*b*};
	G:15 If (|| ≥ *K*)
	G:16 *m' *← *m*_*t*_;
	G:17 **Iterate**(*m'*, *B*, *Result*);
	G:18 For each *r *∈ *Result *with _*r *_= _*m'*_
	G:19 If (*m' *is not maximal w.r.t. *r*) return;
	G:20 *Result *← *Result *⋃ {*m'*};
	}

**Table 2:** Comparing sensitivity of lossy versus lossless compression by Off-line with Extensible Motifs, as applied to real and fake protein families.

File	File len	param density		Lossy	Lossless	Compr ratio %
		K	D			
acnucl	4197	10	3	1717	2425	
	4197	5	3	1757	2370	
acnucl-mix	4149	10	3	1864	2629	+8.4
	4149	5	3	1972	2621	+10.6

gprot	25482	30	3	7003	9879	
	25482	20	3	7399	10276	
gprot-mix	25335	30	3	8179	11945	+20.9
	25335	20	3	8386	11978	+16.5

succ	16297	20	3	4994	7449	
	16297	10	3	4977	7466	
succ-mix	16410	20	3	5929	8803	+18.2
	16410	10	3	6415	8962	+19.2



where (*xy*) denotes the concatenation of *x *and *y*. Hence, *D*(*x*, *y*) measures the improvement over *Off-line*(*y*) that is brought about by using *x *as a "dictionary" when compressing *y*.

In the following experiment we construct a phylogeny of the Eutherian orders using complete unaligned mitochondrial genomes of the following 15 mammals from GenBank: human (Homo sapiens [GenBank:V00662]), chimpanzee (Pan troglodytes [GenBank:D38116]), pigmy chimpanzee (Pan paniscus [GenBank:D38113]), gorilla (Gorilla gorilla [GenBank:D38114]), orangutan (Pongo pygmaeus [GenBank:D38115]), gibbon (Hylobates lar [GenBank:X99256]), sumatran orangutan (Pongo pygmaeus abelii [GenBank:X97707]), horse (Equus caballus [GenBank:X79547]), white rhino (Ceratotherium simum [GenBank:Y07726]), harbor seal (Phoca vitulina [GenBank:X63726]), gray seal (Halichoerus grypus [GenBank:X72004]), cat (Felis catus [GenBank:U20753]), finback whale (Balenoptera physalus [GenBank:X61145]), blue whale (Balenoptera musculus [GenBank:X72204]), rat (Rattus norvegicus [GenBank:X14848]) and house mouse (Mus musculus [GenBank:V00711]).

**Figure 1:**
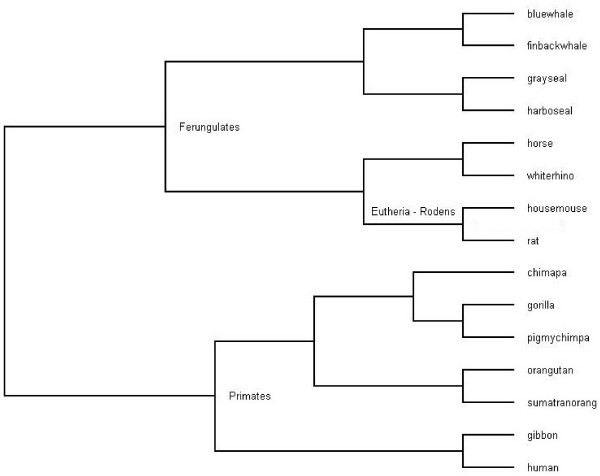
The evolutionary tree built from complete mammalian mtDNA sequences of 15 species. [width = 450 pt]tree1.eps

The evolutionary tree in Figure 1 is generated by a variant of the classical neighbor-join where instead of minimizing the distances between nodes we maximized the separation. Specifically, for each pair (*x*, *y*) of sequences, the quantity *D*(*x*, *y*) is computed. Next, the neighbor-join algorithm is used to build the tree from the matrix of distances. This algorithms selects a pair of (*x*, *y*) among those achieving the minimum value for *D*, and creates an internal node as their father. It then coalesces *x *and *y *into a combined sequence the *D *value of which is computed as the maximum (instead of the average) of those of *x *and *y*. The process is continued until the *D*-matrix has shrunk to a scalar. The first notable finding is that closely related species are indeed grouped together, e.g., grayseal with harboseal, orangutan with sumatranorang, etc. Whereas there is no gold standard for the entire tree, biologists do suggest the following grouping for this case:

• Eutheria-Rodens: housemouse, rat.

• Primates: chimpa, gibbon, gorilla, human, orangutan, pigmychimpa, sumatranorang.

• Ferungulates: bluewhale, finbackwhale, grayseal, harboseal, horse, whiterhino.

The phylogeny obtained in our experiment is very close to the commonly accepted ones, which suggests that even a method of compression based on a single type of regularity, as opposed to those that take into account palindromes and other structures may support good comparative genomics.

**Figure 2:**
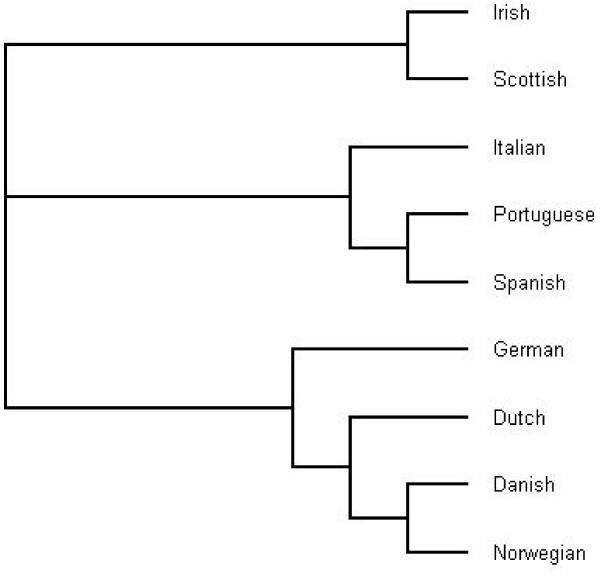
A partial tree of languages using a distance based on compression by extensible motifs. [width = 280 pt]tree2.eps

For a comparison, the same treatment was applied to human language text classification, in analogy with what is found in [7,8]. Figure 2 displays the tree obtained in experiments performed with a small subset of languages on the widely translated "Universal Declaration of Human Rights". Once more, the resulting tree is coherent with commonly accepted ones.

## Conclusion

Comparisons of the compression ratios respectively achieved by rigid and extensible motifs displays that the latter bring about additional savings in compression. This suggests that extensible motifs may be preferred to rigid ones also in those cases where they are used as bases for similarity measure and classification among sequences. The unsupervised classification method built on top of such measures have been shown here to consistently produce phylogenic trees for species genomes as well language classifications built on text documents.

## Methods

### Mining extensible motifs

The procedure of motif extraction that is described in Table 1 essentially constructs the inexact suffix tree of [1] implicitly, in a different order. The input is a string *s *of size *n *and two positive integers, *K *and *D*.

The extensibility parameter *D *is interpreted in the sense that up to *D *(or 1 to *D*) dot characters between two consecutive solid characters are allowed. The output is all maximal extensible (with *D *spacers) patterns that occur at least *K *times in *s*. Incidentally, the algorithm can be adapted to extract rigid motifs as a special case. For this, it suffices to interpret *D *as the maximum number of dot characters between two consecutive solid characters.

The algorithm works by converting the input into a sequence of possibly overlapping *cells*: A cell is the smallest substring in any pattern on *s*, that has exactly two solid characters: one at the start and the other at the end position of this substring. A maximal extensible pattern is a sequence of cells.

#### Initialization phase

The cell is the smallest *extensible *component of a maximal pattern and the string can be viewed as a sequence of overlapping cells. If no don't care characters are allowed in the motifs then the cells are non-overlapping. The initialization phase has the following steps.

Step 1: Construct patterns that have exactly two solid characters in them and separated by no more than *D *spaces or "." characters. This is done by scanning the string *s *from left to right. Further, for each location we store start and end position of the pattern. For example, if *s *= *abzdabyxd *and *K *= 2, *D *= 2, then all the patterns generated at this step are: *ab*, *a.z*, *a..d*, *bz*, *b.d*, *b..a*, *zd*, *z.a*, *z..b*, *da*, *d.b*, *d..y*, *a.y*, *a..x*, *by*, *b.x*, *b..d*, *yx*, *y.d*, *xd*, each with its occurrence list. Thus _*ab *_= {(1, 2), (5, 6)}, _*a.z *_= {(1, 3)} and so on.

Step 2: The extensible cells are constructed by combining all the cells with at least one dot character and the same start and end solid characters. The location list is updated to reflect the start and end position of each occurrence. Continuing the previous example, *b-d *is generated at this step with _*b-d *_= {(2, 4), (6, 9)}. All cells *m *with |_*m*_| <*K *are discarded. In the example, the only surviving cells are *ab*, *b*-*d *with _*ab *_= {(1, 2), (5, 6)} and _*b*-*d *_= {(2, 4), (6, 9)}

#### Iteration phase

Let *B *be the collection of cells. If *m *= *Extract*(*B*), then *m *∈ *B *and there does not exist *m' *∈ *B *such that *m' *∗ *m *holds: *m*_1 _∗ *m*_2 _if one of the following holds: (1) *m*_1 _has only solid characters and *m*_2 _has at least one non-solid character (2) *m*_2 _has the "-" character and *m*_1 _does not, and, (3) *m*_1 _and *m*_2 _have *d*_1_, *d*_2 _> 0 dot characters respectively and *d*_1 _<*d*_2_.

Further, *m*_1 _is ~-compatible with *m*_2 _if the last solid character of *m*_1 _is the same as the first solid character of *m*_2_. Further if *m*_1 _is ~-compatible with *m*_2_, then *m *= *m*_1 _~ *m*_2 _is the concatenation of *m*_1 _and *m*_2 _with an overlap at the common end and start character and _*m *_= {(*x, y*)|(*x, l*) ∈ }. For example if *m*_1 _= *ab *and *m*_2 _= *b.d *then *m*_1 _is ~-compatible with *m*_2 _and *m*_1 _~ *m*_2 _= *ab.d*. However, *m*_2 _is not ~-compatible with *m*_1_.

*NodeInconsistent*(*m*) is a routine that checks if the new motif *m *is non-maximal w.r.t. earlier non-ancestral nodes by checking the location lists. The procedure is best described by the pseudocode shown in Table 1. Steps G:18–19 detect the suffix motifs of already detected maximal motifs. *Result *is the collection of all the maximal extensible patterns.

A tight time complexity for the procedure is not easy to come by, however, if we consider *M *to be the number of extensible maximal motifs and *S *to be the size of the output – i.e. the sum of the sizes of the motifs and the sizes of the corresponding location lists – then the time taken by the algorithm is *O*(*SM *log *M*). In experiments of the kind described later in the paper, at 3 GHz clock, time ranged typically from few minutes to half an hour.

### Compression by extensible motifs

Traditionally, the design of codebooks used in compression proceeds from specifications that are either statistical or syntactic. The quintessential statistical approach is represented by Huffman codes, in which symbols are ranked according to their frequencies and then assigned in order of decreasing probability to longer and longer codewords. In a syntactic approach, the codebook is built out of patterns that display certain features, e.g., of robustness in the face of noise, loss of synchronization, etc. The focal point in these developments is the structure of the codewords. For instance, a codeword is a pattern *w *of length *m *such that any other codeword must be at a distance of *d *from *w*, the distance being measured in terms of errors of a certain type. We can have only substitutions in the *Hamming *variant, substitutions, insertions and deletions in the *Levensthein *variant, and so on. Of course, the two aspects blend in the final code. With Huffmann codes, for instance, once the characters are statistically ranked a code with certain syntactic characteristics, notably, obeying the prefix property, is built. Likewise, once the codebook of an error correcting code is designed, the statistics of the source is taken into account for encoding. However, these two stages are, as a rule, carried out somewhat independently.

The notion of a motif that we adopt tightly combines the structure of the motif pattern, as described by its syntactic specification, with the statistical measure of its occurrence count. This supports a notion of saturation that finds natural use in the dual contexts of structural inference and compression. As said, this saturation condition mandates that motifs that could be made more specific without altering their set of occurrences do not bear interest and may be discarded.

In this Section, we present lossy off-line data compression techniques by textual substitution in which the patterns used in compression are chosen among the extensible motifs that are found to recur in the textstring with a minimum pre-specified frequency. As mentioned, motif discovery and motif-driven parses of various kinds have been previously introduced and used in [5], however, the motifs considered in those studies are "rigid".

The transition from rigid to extensible motifs requires a complete restructuring of the combinatorial and computational tools for their extraction and implementation. Specifically, one needs:

• An algorithm for the extraction of flexible motifs.

• A criterion for choosing and encoding the motifs to be used in compression.

• A new suite of software programs implementing the whole.

The orchestration of these ingredients are briefly described next. We regard the motif discovery process as distributed on two stages, where the first stage unearths motifs endowed with a certain set of properties and the second implements them in the compression. The first part was dealt with in the preceding section. Like with rigid motifs in [5], the flexible ones presented here may be restored at the receiver using information about gap filling, to be transmitted separately. In images, for instance, a tremendous amount of compression is attained, albeit with a large loss such as 40% or so, yet simple predictors in the form of linear interpolation restores more than 95% of the original.

The methods presented here belong to a class of *off-line *textual substitution that try to reap through greedy approximation the benefits of otherwise intractable optimal macro schemes [9]. The specific heuristic followed here is based on a greedy iterative selection (see e.g., [10]) which consists of identifying and using, at each iteration, a substring *w *of the text *x *such that encoding all instances of *w *in *x *yields the highest possible contraction of *x*. This process may be also interpreted as learning a "straight line" grammar of minimum description length for the sourcestring, for which we refer to [5,11,12] and references therein. Off-line methods are not always practical and can be computationally imposing even in approximate variants. They do find use in contexts and applications, such as mass production of CD-ROMs, backup archiving, etc. (see, e.g., [13]). Paradigms of steepest descent approximations have delivered good performances in practice and also appear to be the best candidates in terms of the approximation achieved to optimum descriptor sizes [14].

Our steepest descent paradigm performs a number of phases consisting each in the selection of the pattern to be used for compression followed by the actual substitution and encoding. The process stops when no further compression is achieved. The sequence representation at the outset is finally pipelined into some of the popular encoders and the best one among the overall scores thus achieved is retained. Clearly, at any stage it is impossible to choose the motif on the basis of the actual compression eventually conveyed by that motif. The decision must be based on an estimate, that takes in to account the mechanics of encoding. In practice, we estimate at *log*(*i*) the number of bits needed to encode the integer *i *(we refer to, e.g., [4] for reasons that legitimate this choice). In one scheme [10], one eliminates all occurrences of *m*, and record in succession *m*, its length, and the total number of its occurrences followed by the actual list of such occurrences. Letting |*m*| to denote the length of *m*, *D*_*m *_denotes the number of extensible characters in *m*, *f*_*m *_the number of occurrences of *m *in the textstring, *s*_*m *_the number of characters occupied by the motif *m *in all its occurrences on *s*, |Σ| the cardinality of the alphabet and *n *the size of the input string, the compression brought about by *m *is estimated by subtracting from the *s*_*m *_log |Σ| bits originally encumbered by this motif on *s*, the expression |*m*| log |Σ| + log |*m*| + *f*_*m*_*D*_*m *_log *D *+ *f*_*m *_log *n *+ log *f*_*m *_charged by encoding, thereby obtaining:

*G*(*m*) = (*s*_*m *_- |*m*|) log |Σ| - log |*m*| - *f*_*m*_(*D*_*m *_log *D *+ log *n*) - log *f*_*m*_

This is accompanied by a loss *L*(*m*) represented by the total number of don't cares introduced by the motif, expressed as a percentage of the original length. If *d*_*m *_is the total number of such gaps introduced across all its occurrences, this would be: *L*(*m*) = *d*_*m*_/*s*_*m*_.

Other encodings are possible (see, e.g., [10]). In one scheme, for example, every occurrence of the chosen pattern *m *is substituted by a pointer to a common dictionary copy, and we need to add one bit to distinguish original characters from pointers. The original encumbrance posed by *m *on the text is in this case (log |Σ| + 1)*s*_*m*_, from which we subtract |*m*| log |Σ| + *f*_*m*_*D*_*m *_log *D *+ log |*m*| + *f*_*m*_(log *r *+ 1), where *r *is the size of the dictionary, in itself a parameter to be either fixed a priori or estimated.

## Authors' contributions

All authors contributed equally to this work.
